# Buprenorphine plasma and cerebrospinal fluid concentrations in osteoarthritis patients during low-dose transdermal patch dosing

**DOI:** 10.1007/s00228-023-03583-4

**Published:** 2023-10-21

**Authors:** Lasse Härkänen, Henriikka Hakomäki, Jukka Huopio, Hannu Kokki, Sanni Korhonen, Marko Lehtonen, Sari Sjövall, Merja Kokki

**Affiliations:** 1 School of Medicine, University of Eastern Finland, Kuopio, Finland; 2 School of Pharmacy, University of Eastern Finland, Kuopio, Finland; 3 Department of Orthopedic Surgery, Kuopio University Hospital, Kuopio, Finland; 4 Department of Anestesia, Satakunta Central Hospital, Pori, Finland; 5 Department of Anesthesia and Intensive Care, Kuopio University Hospital, Kuopio, Finland

**Keywords:** Buprenorphine, Transdermal, Cerebrospinal fluid, Plasma

## Abstract

**Methods:**

Fifty-six (56) patients scheduled for arthroplasty, received 7-day extended-release buprenorphine transdermal patches (5 µg/h) for five consecutive weeks, starting two weeks prior to the surgery. Simultaneous plasma and cerebrospinal fluid (CSF) samples were collected during spinal anesthesia.

**Results:**

Median buprenorphine plasma and CSF concentrations at steady-state were 54 pg/mL (range 8.6 – 167 pg/mL) and 1.6 pg/mL (0.30 – 7.3 pg/mL), respectively. The median CSF/plasma -ratio was 3% (range 0.35 – 16%). Large between-subject variability was observed in the measured buprenorphine concentrations within the study population.

## Main

The study was conducted in Kuopio University Hospital, and in Satakunta Central Hospital, Finland. The study protocol was approved by the Research Ethics Committee of the Hospital District of Northern Savo (ref. No. 113/2011), notified to Finnish Medicines Agency (ref. 122//2011), and registered to the European Clinical Trials Database (ref. Eudra CT: 2011–000692-14), as well as to the ClinicalTrials.gov database (ref. NCT02575664, registered 2015–10-09). The clinical trial was conducted in accordance with the Declaration of Helsinki. A written informed consent was collected from the subjects.

Fifty-six patients (23 women, 33 men) with scheduled arthroplasty were enrolled. The subjects were 40–75 years of age. Exclusion criteria were BMI below 18 or over 35 kg/m^2^, ongoing buprenorphine (BUP) treatment, hypersensitivity to transdermal patch ingredients, a history of substance abuse, ongoing monoamine oxidase inhibitor treatment, impairment of renal, hepatic, or pulmonary function, ongoing treatment for constipation, and pregnant or breastfeeding women.

The subjects received BUP through an extended-release transdermal patch, releasing 5 µg/h of BUP (Norspan^®^, Mundipharma Oy, Vantaa, Finland). Five consecutive 7-day patches were administered during the study, starting two weeks prior to the surgery. The subjects were given oral and written instructions to change the patch, to a new skin area, in a 7-day interval. Pain was assessed in a placebo-controlled, randomized, double-blinded trial described in Härkänen et al. [1].

Plasma and CSF samples were collected during spinal anesthesia induction for surgery on the 14th day of BUP exposure, at steady state. CSF sample was collected during lumbar puncture for spinal anesthesia before local anesthetic injection, simultaneously with a venous blood sample.

Buprenorphine concentrations were quantified (as free base) with a sensitive analytical method described in Hakomäki et al. [2]. The lower limit of quantification (LLOQ) in plasma and CSF samples were 1.2 pg/mL and 0.30 pg/mL, respectively.

**Fig. 1:**
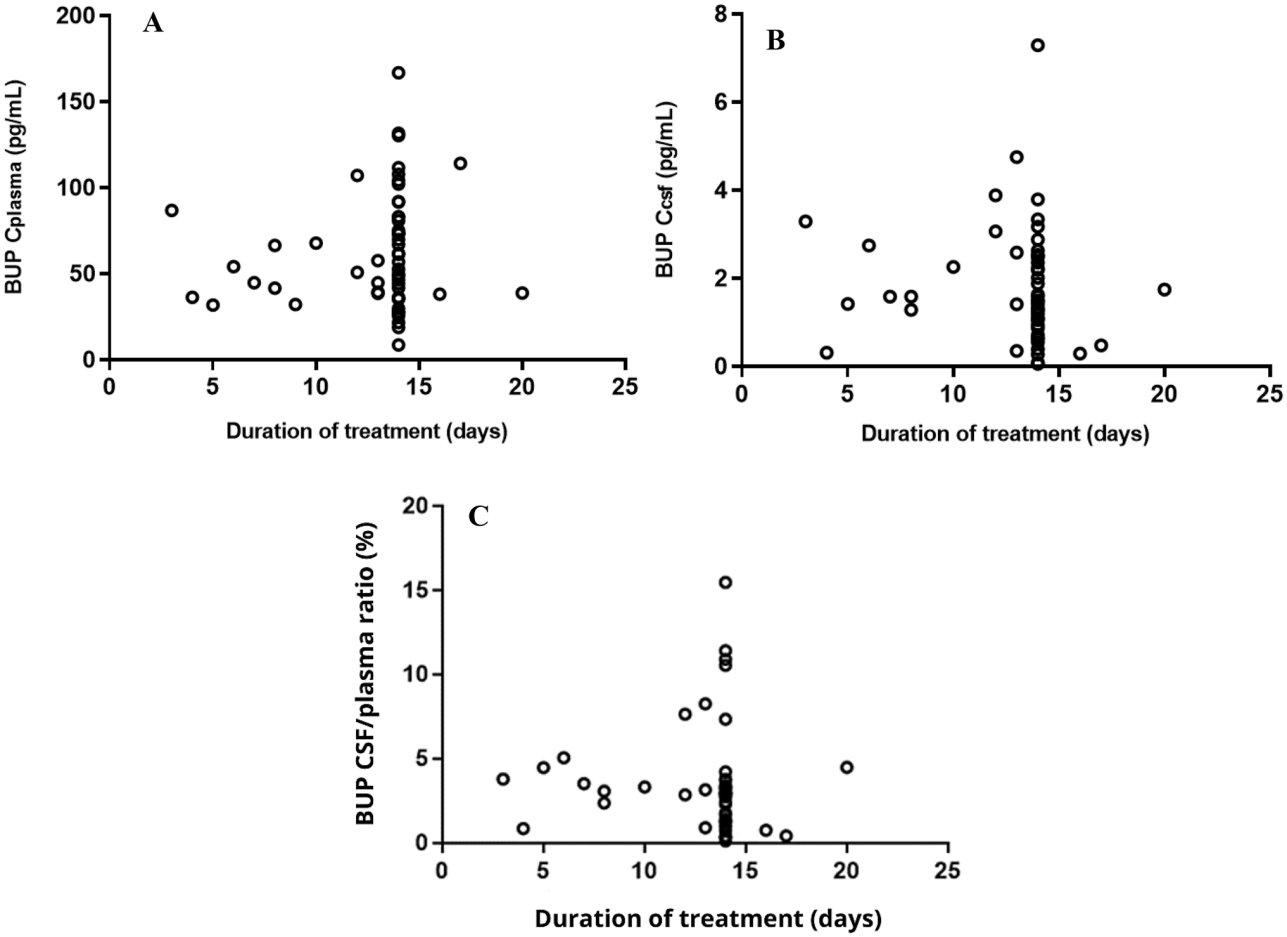
Plasma (figure **A**) and cerebrospinal fluid (CSF, figure **B**) buprenorphine (BUP) concentrations from 56 study subjects receiving 5 µg/h of BUP via extended-release 7-day transdermal patch, and BUP CSF/plasma concentration ratios (figure **C**)

The measured median plasma and CSF BUP concentrations at steady-state were 54 pg/mL (range 8.6–167 pg/mL) and 1.6 pg/mL (0.30–7.3 pg/mL), respectively (Fig. 1A and B). The median CSF/plasma -ratio was 3% (range 0.35–16%) (Fig. 1C). Large between-subject variability was observed in the samples, and in the corresponding CSF/plasma -ratios. Concentrations below the LLOQ were observed in CSF in two subjects.

Apparent plasma clearance (CL_app_) was calculated to try to explain some of the between-subject variability observed. The median CL_app_, calculated by using the infusion rate formula (R_0_ = Plasma clearance * Plasma concentration at steady-state), was 92.4 L/h (range 30–581 L/h). Normalized to body weight, median CL_app_ was 1.13 L/h/kg (range 0.38–7.3 L/h/kg). Our estimate of CL_app_ is similar to the plasma clearance estimated by Jensen et al. [3] during BUP infusion (0.92 L/h/kg). Correlation of CL_app_/kg to age and sex were visually inspected. No correlation was found.

Supporting our data, buprenorphine plasma concentrations measured in the study were similar to those reported by Al-Tawil et al. [4]. Al-Tawil and associates collected plasma samples during 5 µg/h BUP transdermal patch dosing. Consistent with the present study, a mean plasma BUP concentration of 55 pg/mL on the 14th exposure day was reported.

The novelty of the present study is that, to the best of our knowledge, this is the first study to report BUP CSF concentrations in humans. A major strength of the study was the ability to measure plasma and CSF concentrations at steady-state to accurately determine the fraction of BUP able to transfer into the CSF. In plasma, BUP is approximately 96–98% bound to plasma proteins, thus the CSF/plasma -ratio of 3% reported here well represents the unbound fraction of BUP in plasma that is able to cross the blood-cerebrospinal fluid-barrier into CSF.

## Data Availability

Data available upon request.

## Abbreviations

BUP: Buprenorphine
CL_app_: Apparent plasma clearance
CSF: Cerebrospinal fluid
C_csf_: Cerebrospinal fluid concentration
C_plasma_: Plasma concentration
LLOQ: Lower limit of quantification
